# Dynamic Environmental Interactions Shape the Volatile Compounds of Agarwood Oils Extracted from *Aquilaria sinensis* Using Supercritical Carbon Dioxide

**DOI:** 10.3390/molecules30040945

**Published:** 2025-02-18

**Authors:** Wenxian Zhang, Sizhu Qian, Dehuai Wu, Qiaoling Yan, Jen-Ping Chung, Yongmei Jiang

**Affiliations:** 1 College of Life Sciences, Fujian Normal University, Qishan Campus, No. 18 Middle Wulongjiang Avenue, Shangjie, Minhou, Fuzhou 350117, China; wxzhang@fjnu.edu.cn (W.Z.); qiansizhu98@outlook.com (S.Q.); yanql11@outlook.com (Q.Y.); 2 National Quality Supervision and Inspection Center for Incense Products (Fujian), No. 1-1, West Liuan Shan Road, Taocheng Town, Yongchun County, Quanzhou 362600, China; wudehuai1989@outlook.com; 3 College of Horticulture and Landscape Architecture, Fujian Vocational College of Agriculture, No. 116 Guishan, Hongxing Village, Jingyang Town, Fuqing 350119, China

**Keywords:** agarwood oils, supercritical CO_2_ extraction, multivariate statistical technology, metabolomics, GC–MS analysis, chromones

## Abstract

*Aquilaria* spp. are a highly valuable plant species found in the Chinese herbal medicine and agarwood fragrance supplement industries for fumigation, combustion and perfume. The phytochemical composition of agarwood oils (extracts) was derived from *Aquilaria sinensis* and its subspecies ‘Qi-Nan’ using supercritical CO_2_ extraction technology. Gas chromatography connected with a mass spectrometry apparatus was employed for qualitative and quantitative analyses. Comparing the agarwood oils from six planting areas, 12 common components were obtained, among which sesquiterpenes and chromones had the highest relative content. Genetic and environmental factors had the greatest impact on the three chromones, especially on 2-phenyl-4*H*-chromen-4-one. According to the PCA and PLS-DA models, the ‘Qi-Nan’ was derived from a variety selected from the native *A. sinensis*, and the difference in the volatile components was able to indirectly prove that it was genetically heterogeneous with the native *A. sinensis*. Using the 73 components obtained from GC–MS analysis, the VIP values and S-plots were generated using the OPLS-DA model. Seven components with VIP values > 1.0 were selected from two groups of agarwood oils of the native *A. sinensis* and ‘Qi-Nan’ subspecies. In addition, by analyzing 12 common components, the differential components with VIP values > 1 were 2-phenyl-4*H*-chromen-4-one and 2-(4-methoxyphenethyl)-4*H*-chromen-4-one. Chromones were the main component of agarwood oils extracted by supercritical CO_2_, and 2-phenyl-4*H*-chromen-4-one could be used as a volatile marker, especially in the ‘Qi-Nan’ subspecies, where this marker exhibited more prominent characteristics.

## 1. Introduction

Agarwood is a resin containing wood parts formed by plants in the *Aquilaria* genus of the family Thymelaeaceae under natural or artificial interventions. It has an aroma and is widely used in the treatment of arthritis, asthma, rheumatism, gout and other diseases [1,2]. *Aquilaria* spp. are mainly distributed in Asian countries such as India, Myanmar, Laos, Vietnam, Cambodia, Thailand and China. The three species of agarwood trees, *A. malaccensis*, *A. crassna* and *A. sinensis*, are the main strains of agarwood in the market [3,4]. In China, with the diversification of fragrance, a new variety with excellent characteristics has been selected from the natural forests of native *Aquilaria sinensis*. These subspecies have the advantages of early production and fast speed for the resin formation, and high fragrance quality, namely ‘Qi-Nan’, and has been widely planted in southern China [5].

In addition to being used as a Chinese herbal medicine, agarwood fragrance products such as agarwood essential oils, agarwood oils (extracts), agarwood powder and agarwood incense, are often used for fumigation and combustion, playing a role in soothing the body and mind and relieving depression [6]. The extraction methods of agarwood oils (AOs) include solvent extraction, steam distillation and supercritical CO_2_ fluid extraction (SFE-CO_2_), which are more common. Traditionally, perfume in the Middle East uses agarwood essential oils hydro-distilled from the fragrant part of the *Aquilaria* tree. However, the SFE-CO_2_ method can not only carry out separation operation at a lower temperature to ensure that the aroma components are not destroyed but also use the special solubility of critical CO_2_ to obtain more volatile components. It has obvious advantages in saving energy and can obtain differentiated quality from aromatic oil products by steam distillation, microwave-ultrasonic methods and hydro-distillation [7]. The SFE-CO_2_ extraction of AOs has good application value in economic yield, aroma adaptability and biotechnology processing applications, and is the most used extraction method in China [8,9]. The main components are sesquiterpenes, 2-(2-phenylethyl) chromones, lipids and aromatic compounds [10,11,12]. However, the tree species, planting area, resin formation methods and extraction methods affect the components of agarwood. Studies suggest that the quality of AOs with a high content of chromones is better [13,14]. Chromone components can be used as characteristic indexes for quality evaluation and the identification of agarwood.

The composition of natural substances varies with plant species and the cultivation environmental factors, and the quantitative data between dynamic environments cannot be obtained absolutely by analytical instruments. In this study, native *A. sinensis* in China, is mainly concentrated in Guangdong, Guangxi and Hainan areas, while the volatile components of the AO in Taiwan have not been reported. The acquisition of wild agarwood is a high price and appears in the Appendix II list of The Convention on International Trade in Endangered Species of Wild Fauna and Flora, since 2004. Artificial agarwood products will be the trend of the future market. We collected the artificial agarwood parts of the agarwood trees in different regions of South China and obtained the agarwood oil samples by SFE-CO_2_ for GC–MS analysis. Multivariate statistical techniques including principal components analysis (PCA), partial least squares discriminant analysis (PLS-DA) and orthogonal partial least squares discriminant analysis (OPLS-DA) were utilized to find the different chemical components of AOs from different plantings and genetic varieties [15,16,17]. NIST database retrieval and literature reviews were used to identify different chemical components, providing references for quality assessment and the functional components of AOs.

## 2. Results

### 2.1. Analysis of Volatile Components in AOs by SFE-CO_2_

We analyzed the volatile components of the AOs extracted by SFE-CO_2_ using GC–MS linked to NIST 2020 database, as shown in Table 1.

**Figure 1:**
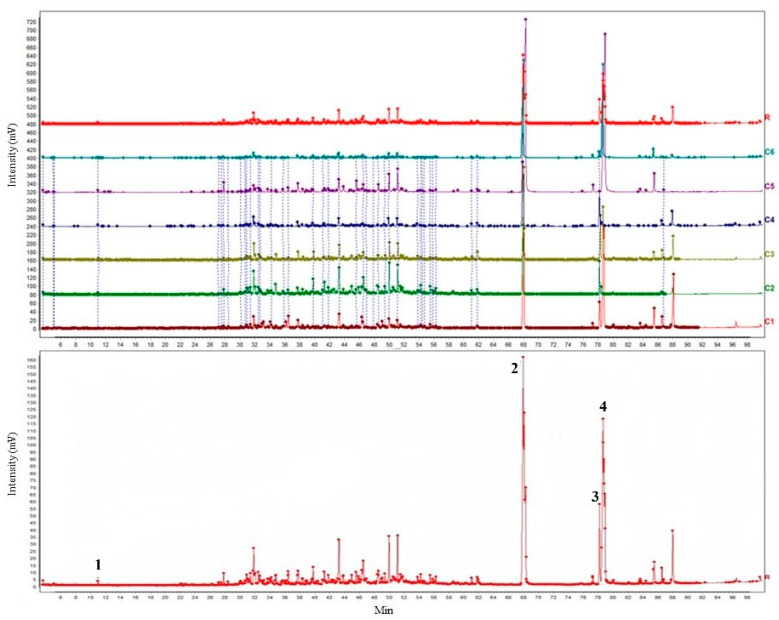
Total ion chromatogram (**above**) and control spectrum (**below**) of AOs extracted by SFE-CO_2_. Mark 1: 2-butanone, 4-phenyl-; Mark 2: 2-phenethyl-4*H*-chromen-4-one; Mark 3: 6-methoxy-2-phenethyl-4*H*-chromen-4-one; Mark 4: 2-(4-methoxyphenethyl)-4*H*-chromen-4-one.

The 2-(2-phenylethyl) chromone compounds were determined based on the mass spectrometry characteristics and fragmentation patterns summarized in the literature [10,18], combined with the characteristics of ion fragments in this study (Figure 1). According to the NIST 2020 database, more than 85% of similar components were compared. A total of seventy-three substances were identified, among which the main components were forty-three sesquiterpenes, twelve aromatic compounds, six chromones and one triterpene (Table 1). Comparison of the common components in AOs of the C1–C6 planting areas revealed a total of 12 components, with the relative content of sesquiterpenes and chromones being the majority. The common components of the bold font indicated in Table 1 showed three aromatic compounds: benzaldehyde (CAS 100-52-7), 4-phenyl-2-butanone (CAS 2550-26-7) and 1,5-diphenyl-1-en-3-one (CAS 62510-08-1); five types of sesquiterpene compounds: santalol (CAS 11031-45-1), (2*R*)-2,3,4,4a, 5,6,7,8-Octahydro—α, α, 4a β, 8 β-tetramethyl-2-naphtholemethanol (CAS 63891-61-2), agarosol (CAS 1460-73-7), 2(3*H*)-naphtholene, (4aR, 5S)- 4a, 5-dimethyl-3-propan-2-ylidene-5,6,7,8-tetrahydro-4*H*-naphthol-2-one (CAS 19598-45-9) and arctiol (CAS 36061-11)-7); and three types of 2-(2-phenylethyl)chromones: 2-phenyl-4*H*-chromen-4-one (CAS 61828-53-3), 6-methoxy-2-pheneth-4*H*-chromen-4-one (CAS 84294-89-3) and 2-(4-methoxyphenyl)-4*H*-chromen-4-one (CAS 92911-82-5). In addition, a triterpene squalene (CAS 111-02-04) was analyzed, indicating that these 12 components were representative of the AOs extracted by SFE-CO_2_. For the agarwood extracts sampled in this study, GC–MS data showed that 4-phenyl-2-butanone was the specific index component of agarwood, showing that three functional chromone components of agarwood were obtained, which could prove that the agarwood oil samples were determined to be agarwood (Figure 1).

### 2.2. Genotype and Environmental Factors Have the Greatest Influence on Chromone Components

**Figure 2:**
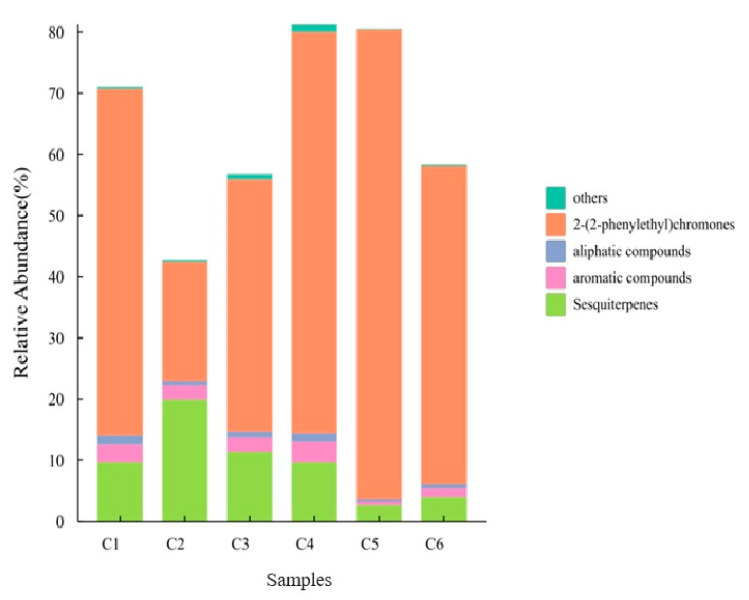
Overlay diagram of the main composition categories of AOs extracted by SFE-CO_2_. Sample C1: Nantou, Taiwan; C2: Maoming, Guangdong; C3: Zhuhai, Guangdong; C4: Nanning, Guangxi; C5: Huizhou, Guangdong; C6: Huidong, Guangdong. Data were analyzed by the public resource ChiPlot website (https://www.chiplot.online, accessed on 24 February 2024).

**Figure 3:**
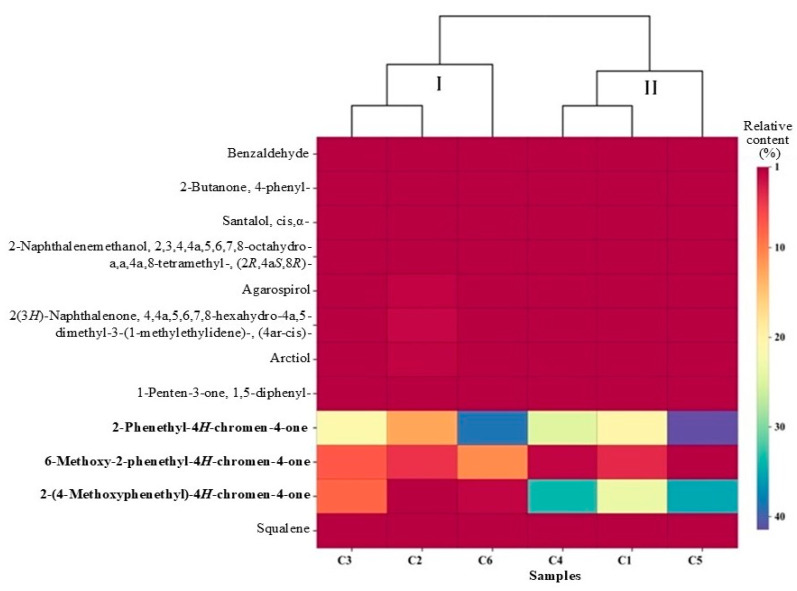
The clustering heatmap of the 12 common components of AOs extracted by SFE-CO_2_. The red to blue bar icon on the right shows the relative content. The bold font on the left represents three types of chromones. Sample C1: Nantou, Taiwan; C2: Maoming, Guangdong; C3: Zhuhai, Guangdong; C4: Nanning, Guangxi; C5: Huizhou, Guangdong; C6: Huidong, Guangdong. Data were analyzed by the public resource ChiPlot website (https://www.chiplot.online, accessed on 24 February 2024).

Due to the complexity of the factors affecting the compositions of the plant metabolites, to gain an understanding of the huge dataset acquired from GC–MS analysis, the online public resource ChiPlot website (https://www.chiplot.online, accessed on 24 February 2024) was used to draw an overlay diagram of the main composition categories of the AOs (Figure 2) and a clustering heatmap of 12 common components (Figure 3). Visual results showed that the AOs of the C1–C6 planting areas were mainly composed of chromones, with the total relative content between 42.8% and 81.3%, of which chromones were between 19.3% and 76.8% and sesquiterpenes were between 2.7% and 19.9% (Figure 2). Chromones and sesquiterpenes were also the most important composition types in other research reports about agarwood. The relative sesquiterpene content of the C2 region was highest at 19.9%, and the C5 region had the lowest content but the chromones were the highest at 76.8%. However, the chromone content of the C2 region was the lowest at 19.34% (Figure 2). For the relative content of sesquiterpenes in the C5 and C6 regions, *A. sinensis* ‘Qi-Nan’ subspecies had significantly lower levels than that of the native *A. sinensis* species (C1–C4). The total relative content of chromones in C5 of the ‘Qi-Nan’ subspecies was the highest. However, the total relative content of chromones in C6 of the ‘Qi-Nan’ subspecies was lower than that of *A. sinensis* in both C1 (Taiwan) and C4 (Guangxi). It was difficult to obtain the impact results between the different environments and the two varieties from the data in Figure 2, so we roughly summarized preliminary data that were obtained of the chromones and sesquiterpenes in agarwood.

Further visualization of the 12 common components in the C1–C6 planting areas are compared in Table 1 (black bold font), and the 12 common components were divided into groups I and II: C2, C3, C6 and C1, C4, C5 (Figure 3). The statistical results of the analysis based on the relative content of common components showed that the same ‘Qi-Nan’ subspecies or the planting areas were not divided into one category. The common components of AOs did not form a specific category with the varieties and regions. However, for the three common components of the chromones, 2-phenyl-4*H*-chromen-4-one, 6-methodyl-2-phenyl-4*H*-chromen-4-one and 2-(4-methoxyphenyl)-4*H*-chromen-4-one had different color changes in the clustering heatmap of the three chromones, and the remaining common components had little change (Figure 3). Through the visualization of the clustering heatmap, it was shown that genetic and environmental factors had the greatest impact on the three chromones, especially the 2-phenyl-4*H*-chromen-4-one component. Under different genotypes and environmental conditions, the relative content of the sesquiterpenes was low and the changes were not significant. For example, agarospirol, which is commonly found in agarwood, could not be used as an indicator component for evaluating quality.

### 2.3. Chemometrics Analysis of AOs by SFE-CO_2_

#### 2.3.1. Discrimination Based on PCA and GC–MS Data Fusion

**Figure 4:**
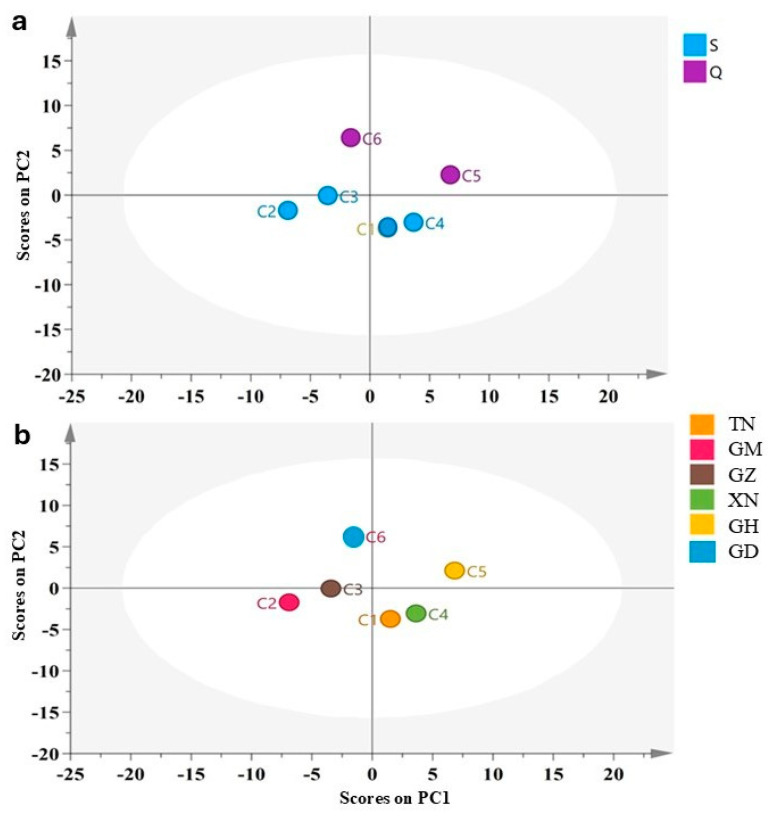
PCA score chart of *Aquilaria sinensis* (blue) and its subspecies ‘Qi-Nan’ (purple) (**a**), and PCA score chart of six planting regions in China (**b**). C1: Nantou, Taiwan (TN); C2: Maoming, Guangdong (GM); C3: Zhuhai, Guangdong (GZ); C4: Nanning, Guangxi (XN); C5: Huizhou, Guangdong (GH); C6: Huidong, Guangdong (GD). Two-dimensional PCA score plot (PC1 vs. PC2) of all chromatographic fingerprints as listed in Table 1.

The GC–MS data of plant secondary metabolites often change with varieties, planting environment and processing methods, so it is difficult to scientifically compare the principal contribution components of the AOs. The 73-component data of the AOs were imported into SIMCA 14.1 software and analyzed by the unsupervised PCA method. The classification was divided into *A. sinensis* (S) and its subspecies ‘Qi-Nan’ (Q) and generated two principal components and a PCA score map (Figure 4a). Under the classification of the two varieties, the PC1 contribution value was 49.1%, the PC2 contribution value was 28.2% and the cumulative contribution rate was 77.3%, indicating that the two principal components were 77.3% of the GC–MS analysis, which was consistent with the model prediction. In addition, PCA analysis was classified according to the six planting areas and a PCA score chart was generated (Figure 4b). According to the two analysis results from PCA, the volatile components of the same variety and subspecies were clearly distinguished (Figure 4a), and further, the same two species of agarwood oil sources were consistent with the specific regions (Figure 4a,b). This indicated that although the genotype had an impact on the volatile components, the planting area was the main factor. The same subspecies of ‘Qi-Nan’ (C5 and C6) also had differences in different planting areas and could not maintain their similar volatile composition characteristics.

Using the visual model of PCA and GC–MS data fusion, the analysis results of the agarwood oil extracted by SFE-CO_2_ clearly distinguished the four quadrants in the PCA model (Figure 4), that is, both the factors of different planting regions and varieties generated significant differences in the volatile components. The results shown in Figure 4 combined with those in Figure 2 illustrated that PC1, which contributed significantly to C1, C4 and C5, were positive scores, and the two important components of chromones and sesquiterpenes were more prominent, especially in the C5 sample, which had higher scores than the origin scores in PC1 and PC2. Therefore, the numerous values obtained from GC–MS could be used to evaluate the quality of agarwood oil through PCA multivariate statistical analysis.

#### 2.3.2. PLS-DA and OPLS-DA Models Distinguished Genetic Heterogeneity

**Figure 5:**
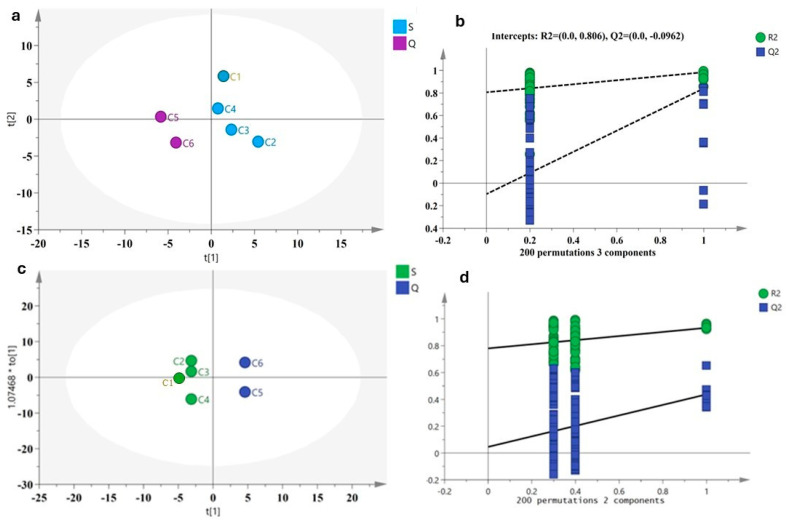
Scatter plots of PLS-DA and OPLC-DA were obtained from the classification of AOs between the native *A. sinensis* (S) and ‘Qi-Nan’ subspecies (Q). (**a**,**b**) PLS-DA scatter plot, and the verification results of 200 permutations; (**c**,**d**) OPLS-DA scatter plot, and the verification results of 200 permutations. R2: the coefficient of determination, Q2: squared cross-validation. The Hotelling’s T2 analysis showed that all samples were within the 95% confidence interval.

PCA is an unsupervised chemometrics analysis method, which performs dimensionality reduction transformation on GC–MS data, and linearly classifies the feature vectors after dimensionality reduction to obtain the descriptive map regions of different germplasm resources and components [19]. However, the intra-group differences and random errors cannot be ignored when determining the components of differences. Therefore, based on PCA, this study used the supervised methods PLS-DA and OPLC-DA to determine the different volatile components of AOs from the six planting areas and the two varieties of native *A. sinensis* and ‘Qi-Nan’ subspecies. Using the data from GC–MS, the classification was divided into *A. sinensis* (S) and subspecies ‘Qi-Nan’ (Q) according to the provenance. In the PLS-DA model, there was a clear separation between the six samples (regions) and the two genotypes when assessing the AOs (Figure 5a). The SIMCA14.1 software automatically generated the values R^2^X (cum) = 0.897, R^2^Y (cum) = 0.957 and Q^2^ (cum) = 0.718, indicating that 89.7% of the peak independent variables could explain 95.7% of the dependent variables of the two classifications of genetic varieties. The threshold Q^2^ > 0.5 indicated that the prediction ability was strong. The results showed that there were significant habitat differences in the distribution of volatile components. It is worth noting that the trees of the native *A. sinensis* were distributed in the quadrant on the right regardless of the origin, while the ‘Qi-Nan’ subspecies were distributed in the quadrant on the left (Figure 5a). Discrimination of different genotypes had a certain effect. The permutation validation in SIMCA 14.1 software was used to verify the fitting of PLS-DA (Figure 5b). Through 200 iterations of permutation testing, the model results showed that the *Y*-axis intercepts were all less than 0, indicating that the PLS-DA model validation results were fitting and reliable. Alternatively, the Hotelling’s T2 analysis also verified that all samples were within the 95% confidence interval [20,21]; these validation results provided a more robust evaluation of model performance.

Further, the supervised OPLS-DA method was used to analyze the differences of the AOs extracted by SFE-CO_2_ from the native *A. sinensis* and ‘Qi-Nan’ subspecies from different planting areas in China, and to screen the volatile markers of AOs. In the OPLS-DA scatter plot (Figure 5c), the R^2^X (cum) = 1, R^2^Y (cum) = 1 and Q^2^ (cum) = 1. The samples were located on both sides of the positive and negative axes of the first principal component with the *X*-axis at 0. These results indicated that the volatile components of the AOs from the native *A. sinensis* could effectively distinguish between the two quadrants of the ‘Qi-Nan’ subspecies. Even if the tree was planted on the island of Taiwan far away from the Chinese mainland, it also showed the same genetic clustering of the *A. sinensis* species on the OPLS-DA (Figure 5c). The 200 permutation tests were conducted to verify the OPLS-DA model and the results showed that the model was reliable (Figure 5d); and the Hotelling’s T2 analysis showed that all the samples were within the 95% confidence interval. Figure 5c revealed that the ‘Qi-Nan’ was derived from a variety selected from the native *A. sinensis*, and the difference in the volatile components were indirectly able to prove that it was genetically heterogeneous with the native *A. sinensis*.

#### 2.3.3. OPLS-DA Model Screening of the Volatile Markers of AOs

**Figure 6:**
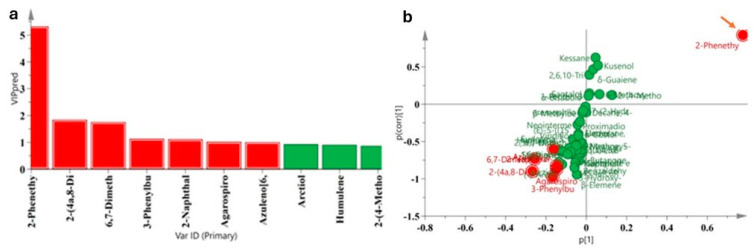
VIP value diagram (**a**) and S-plot diagram (**b**) of the AOs were obtained by the OPLS-DA model. (**a**) The OPLS-DA model showed that there were seven component markers (red color) with a VIP value > 1, the seven components of red dots are shown in Table 2. (**b**) The largest *P*(corr) value was 2-phenethyl-4*H*-chromen-4-one (red arrow) and far away from the origin and the main compound group, which contributed the most to the marker.

The VIP value and S-plot evaluation methods were used to find the target components that contributed the most to the groupings of the AOs. The variables with a VIP value greater than one were considered statistically significant and were important markers of the model. The S-plot is a scatter plot that combines the covariance and correlation loading profiles resulting from an OPLS-DA model [22]. The *P*(corr) value was used to explain the contribution of the components to the samples. The further away from the origin and the main compound group, the greater the contribution to the components. In this study, the VIP values (Figure 6a) and S-plot (Figure 6b) were generated by the OPLS-DA model, showing that there were seven components in the two groups of the native *A. sinensis* and ‘Qi-Nan’ subspecies AOs with a VIP value > 1.0 (Table 2).

**Figure 7:**
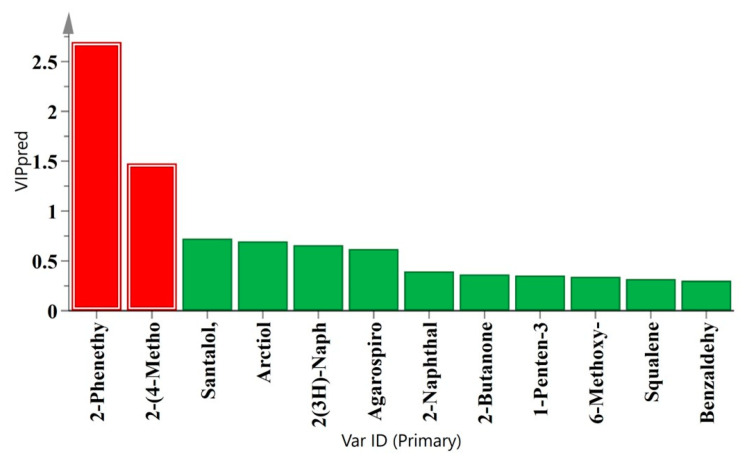
The VIP values of 12 common components were obtained by OPLS-DA between the native *Aquilaria sinensis* and its subspecies ‘Qi-Nan’. There were 2 component markers (red color) with a VIP value > 1.

The component with the largest contribution value was 2-phenethyl-4*H*-chromen-4-one (VIP = 5.32), which was positively correlated with the groupings of AOs by SFE-CO_2_ (Figure 6b; Table 2), while the other six differential components were positively correlated with the groupings. In addition, the 12 common components were analyzed by the OPLS-DA model (Figure 3 and Figure 7). The differential components with a VIP value > 1 were 2-phenyl-4*H*-chromen-4-one (VIP = 2.70) and 2-(4-methoxyphenethyl)-4*H*-chromen-4-one (VIP = 1.48). In conclusion, chromones were the main components of the AOs extracted by SFE-CO_2_, and 2-phenethyl-4*H*-chromen-4-one can be used as the marker of chemical fingerprint, especially as this marker showed more prominent characteristics in the ‘Qi-Nan’ subspecies.

## 3. Discussion

In a natural environment, agarwood resin only occurs when it is subjected to specific environmental stresses, such as lightning strikes, fires, animal grazing, insect attacks or microbial invasion, typically around damaged or decaying parts of the trunk [23]. The artificial intervention method developed to produce agarwood aims to improve the yield and quality, both in terms of raw materials and oil, which will be the future trend in the production of resinous agarwood [24]. Therefore, the plant complexity of the AOs means that a large amount of plant–fungus-mediated secondary metabolites is produced as chemical signals for natural ecological communication [12,25,26]. GC–MS is considered a mature and reliable method for analyzing plant extracts. In this study, AOs extracted by SFE-CO_2_ revealed the differences among sesquiterpenes, chromones and their derivatives, and low molecular weight aromatic compounds extracted from the native *A. sinensis* and its subspecies ‘Qi-Nan’ (Table 1; Figure 2). The quality identification of components was directly compared with the data from analytical instruments, and the 12 common components were obtained through sample comparison (Table 1; Figure 3). However, metabonomic data typically contain a large dynamic range of metabolite concentrations, particularly influenced by variety, planting environment and processing, and it was difficult to obtain the representative components due to variations in variety and environmental factors [3]. Therefore, through a series of statistical models, we could look at the scientific analysis results [13,19,27].

In spiritual practices, agarwood has traditionally been used as a spice and aromatic agent. Agarwood extracts, either essential oils or agarwood oils (or agarwood extracts), can also be used as supplementary medicinal materials, which have antioxidant, antibacterial, anti-inflammatory, air purifying, relaxing and calming properties [28]. Among the two major categories, chromones and sesquiterpenes, mentioned in the research literature of *Aquilaria* spp., the relative content of chromones from agarwood oils extracted by SFE-CO_2_ was relatively high, especially in ‘Qi-Nan’ (Table 1 and Figure 2). Through CO_2_ supercritical fluid extraction and microwave-assisted extraction, the reported agarwood extracts usually contain flidersiachromone, 6-methoxy-2-(2-phenylethyl) chromone, 4*H*-1-benzopyran-4-one chromone and a few other semi-volatile 2-(2-phenylethyl) chromones [14,18]. However, in the water-distilled agarwood essential oil, 2-(2-phenylethyl)chromones often do not appear due to the high temperature during the extraction process [14]. According to modern pharmacology, chromones have been isolated and found to have 240 different subunits. They have anti-inflammatory and anti-tumor properties, neuroprotective effects and inhibitory effects on acetylcholinesterase, tyrosinase, and glucosidase [29]. The three types of 2-(2-phenylethyl)chromones, 2-phenyl-4*H*-chromone, 6-methoxy-2-phenyl-4*H*-chromone and 2-(4-methoxyphenyl)-4*H*-chromone, had common components in the SFE-CO_2_ extracts (Figure 1). It is worth noting that a triterpenoid squalene was analyzed, which was originally extracted from shark liver oil. Its properties were not easily affected by lipid peroxidation and provided skin protection for the human body. It has been studied as an adjuvant therapy for cancer (DrugBank Online-Squalene: https://go.drugbank.com/drugs/DB11460, accessed on 17 January 2025). We have not yet found this in any of the literature related to agarwood, but were able to find it in the AOs extracted by SFE-CO_2_ in this study.

Sesquiterpene components of agarwood have good biological activity in the central nervous system, respiratory system and digestive system [30,31]. In the fungus-mediated fermentation of resinous agarwood, the most significant finding was the appearance of key agarwood sesquiterpenes such as agarospirol, γ-eudesmol and (−)-aristolene [32]. In this study, visual results showed that the components of agarwood oil in native *A. sinensis* and ‘Qi-Nan’ subspecies were similar, but the component numbers of sesquiterpenes and chromones were identified in *A. sinensis* was increased (Table 1; Figure 2). Visual analysis of the twelve common components showed that the relative content of three chromones changed significantly in different planting areas, and the common components of other types were relatively stable (Figure 3). In particular, the relative content of 2-phenyl-4*H*-chromone was higher in ‘Qi-Nan’. Even if these were planted in different environments, the difference in the chemical fingerprint would still exist, which can infer that the two varieties have been genetically separated.

In this study, for the 12 common components of the AOs from different regions, PCA, PLS-DA and OPLC-DA methods were used for multivariate statistical analysis to identify the quality markers [19,32]. Through the visual model of PCA and GC–MS data fusion, the analysis results of the agarwood oil extracted by SFE-CO_2_ clearly distinguished the four quadrants (Figure 4). In the PCA model, PC1, which had a significant contribution, was the sample with a positive score. The two important components of chromones and sesquiterpenes were more prominent (Figure 2 and Figure 4), especially the C5 ‘Qi-Nan’ sample, which scored higher than the original scores of PC1 and PC2. The PCA model effectively distinguished volatile components between the genotypes and origins, and can be applied to the quality identification of fruit aroma and traditional Chinese medicine [33]. Prediction and descriptive modeling, as well as selecting discriminative variables, determined the chemical compositions from different genotypes and product areas, and automatically generated more important principal components [19,34]. According to the PLS-DA and OPLS-DA models (Figure 5), the ‘Qi-Nan’ was derived from a variety selected from the native *A. sinensis*, and the difference in the volatile components indirectly proved that it was genetically heterogeneous with the native *A. sinensis* [35].

The large number of samples analyzed by GC–MS can result in a tedious and time-consuming task for analyzing the output data. However, GC–MS combined with chemometric analysis may be an effective method for studying volatile compounds in AOs. By using OPLS-DA for VIP screening, the contribution of each variable to the classification can be quantified [15]. Scientific statistical screening of the two chromone indicator components, 2-phenyl-4*H*-chrome-4-one and 2-(4-methoxyphenethyl)-4*H*-chrome-4-one, was performed from 73 components (Figure 6 and Figure 7). Due to the stable presence of these two chromone components in the AOs extracted by SFE-CO_2_, especially 2-phenyl-4*H*-chrome-4-one, they can serve as markers for chemical fingerprint identification, and this particular marker exhibited more prominent characteristics in the ‘Qi-Nan’ subspecies. The 2-phenyl-4*H*-chromone could also be used in the design of specific antihistamine drugs [36].

## 4. Materials and Methods

### 4.1. Agarwood Extracts

*Aquilaria sinensis* and *Aquilaria sinensis* ‘Qi-Nan’ originated from six planting areas such as Guangxi, Guangdong and Taiwan in areas of China. The quasi-symbiotic bacteria A329 derived from *A. malaccensis* agarwood had *Bacillus*-like characteristics, was low in phytopathogens [26], and could coculture with *A. sinensis* with the current designed in vitro culture medium. In June 2022, the A329 strain was injected into 6–10 y agarwood trees using artificial induction technology, and after 18 months of induction, agarwood resin was formed. The resinous parts of different trees were randomly obtained from each area and mixed into 2–3 kg shatters, which were then transported to the National Quality Supervision and Inspection Center for Incense Products (Fujian), China, for supercritical CO_2_ fluid extraction (SFE-CO_2_). The agarwood extracts were obtained through SFE-CO_2_. See Table 3 for the source information.

#### Supercritical CO_2_ Extraction Method

According to Dehui Wu and co-workers’ investigation, the supercritical fluid CO_2_ extractor (Hoili biotechnology Inc., HLSFE-12L*2, Guangzhou, China) was connected with supercritical CO_2_. The naturally dried, powdered resin portion (2 kg) of the agarwood plant materials were placed into a stainless-steel extractor container, where the temperature was adjusted to 65 °C, the extraction kettle pressure was set at 40 MPa, and the separation kettle pressure was set at 20 MPa. The flow rate of CO_2_ was 100 L/h and the circulating pressure was 16 MPa.

### 4.2. GC–MS Analyses of AOs

#### 4.2.1. Preprocessing

A total of 30 mg of the AOs was accurately weighed into a 5 mL EP tube, then 2 mL of ethyl acetate solution (China National Pharmaceutical Group Chemical Reagent Co., Ltd., Chengdu, China) was added to dissolve the AOs, then shaken well, and stood for 2 h. To extract, 1mL of the oil solution was filtered through a 0.45 µm filter membrane, preparing it for gas chromatography–mass spectrometry analysis. The diluted agarwood oil extracted above was sampled three times per source region.

#### 4.2.2. GC–MS Analysis

The compositions of the AOs were analyzed by the GCMS-QP2010 Plus (Shimadzu, Tokyo, Japan), equipped with an SH-Rxi-5Sil MS Cap. column (30 m × 0.25 mm i.d., 0.25 μm film thickness; Shimadzu, Tokyo, Japan). The temperature program was as follows: initial temperature 90 °C for 2 min, then increased by 2 °C min^−1^ to 150 °C and held for 5 min, and then increased by 2 °C min^−1^ to 280 °C and held for 5 min. The other parameters were as follows: injection temperature, 250 °C; ion source temperature, 230 °C; EI, 70 eV; carrier gas, He at 1 mL min^−1^; injection volume, 1 μL; spilt ratio, 1:20; and a solvent delay of 2.5 min and mass range, *m*/*z* 50–550. Quantification was obtained for the percentage peak areas from the gas chromatogram. The identification of individual compounds was carried out using the NIST2020 (National Institute of Standards and Technology, US. Department of Commerce) Registry of Mass Spectral Database to search the compounds for authentic references. Chromatographic results, expressed as area percentages, were calculated with a response factor of 1.0.

Each experiment was repeated three times. Based on the NIST2020 database, the volatile components of the samples were qualitatively analyzed by mass spectrometry. Substances with a similarity greater than 85% were identified as potential chemical components of AOs.

### 4.3. Methodological Examination

#### 4.3.1. Precision Test

In the C1–C6 planting areas of the AOs from different sources (Table 1), one region was randomly selected, such as C1. Out of the five samples in each region, equal amounts were drawn and thoroughly mixed to form one sample. The test solution was prepared according to the above preprocessing description, and GC–MS analysis was conducted under the above chromatographic and mass spectrometric conditions. Following the same process, the analysis was repeated six times on the mixed C1 sample. The six data points were compared using the Similarity Evaluation System for the Chromatographic Fingerprint of Traditional Chinese Medicine (Version 2012) [37], and a similarity of no less than 0.99 indicated fine precision of the instrument.

#### 4.3.2. Repeatability Test

For the repeatability test, samples of the AOs from the same source (such as C1) were used. Five samples were made according to the steps described above. The weighing of each sample had to be precise. GC–MS analysis was conducted as described above. The six data points were compared using the Similarity Evaluation System for the Chromatographic Fingerprint of Traditional Chinese Medicine, and a similarity of no less than 0.99 indicated good repeatability of the method.

#### 4.3.3. Stability Test

Any sample solution from C1–C6 was randomly selected, and the selected sample (such as C1) was dissolved into a tube of solution following the aforementioned preprocessing steps. The solution was stored for different times: 2, 4, 6, 8, 12 and 24 h for GC–MS analysis. The six data points were compared using the Similarity Evaluation System for the Chromatographic Fingerprint of Traditional Chinese Medicine, and a similarity of no less than 0.99 indicated that the test solution was stable within 24 h.

#### 4.3.4. Data Processing

Each experiment was repeated three times. Based on the NIST2020 database, the volatile components of the samples were qualitatively analyzed by mass spectrometry. Peak area normalization was used to calculate the relative percentage content. Substances with a similarity greater than 85% were identified as potential chemical components of the AOs. Using the software of Similarity Evaluation System for Chromatographic Fingerprint of Traditional Chinese Medicine (Version 2012) [37] with a time window of 0.2, automatic matching was performed through multi-point correction using the median method. The similarity and common peaks between each sample and the reference map were calculated, and a GC–MS fingerprint map was constructed.

### 4.4. Multivariate Analysis

The SIMICA14.1 software (Umetrics Co., Umeå, Sweden) for multivariate data analysis was used. The compound data were normalized, and then the software was used to perform multivariate statistical analysis through the PLS-DA analysis and OPLS-DA modules. PLS-DA and OPLS-DA were introduced for the discrimination and derivation of potential markers (VIP score >1). Finally, the cluster analysis was carried out in combination with SPSS27.0 data processing software. The univariate statistical analysis was introduced to confirm those differentially expressed features (*p* < 0.05). The mapping software used was the public resource ChiPlot (https://www.chiplot.online, accessed on 24 February 2024). The cluster analysis used between-cluster linkage, and the Euclidean distance was used as a sample measure to determine the differences between the producing regions and the species of AOs.

## Figures and Tables

**Table 1:** Chemical compositions of supercritical CO_2_ extracted agarwood oils analyzed by GC–MS.

NO.	Notes	Compounds	MW	MF	Relative Content (%)					
					C1	C2	C3	C4	C5	C6
**1**	*	**benzaldehyde**	**106**	**C_7_H_6_O**	**0.02**	**0.12**	**0.09**	**0.16**	**0.06**	**0.08**
2		decane, 4-methyl-	156	C_11_H_24_	-	0.02	-	-	0.01	-
3	*	salicylaldehyde	122	C_7_H_6_O_2_	0.02	0.02	0.03	-	-	-
4		undecane, 5,7-dimethyl-	184	C_13_H_28_	-	0.03	-	0.08	0.01	-
5		nonane, 5-(2-methylpropyl)-	184	C_13_H_28_	0.01	0.03	0.01	-	-	-
6		2,6,10-trimethyl-dodecan	212	C_15_H_32_	0.01	0.02	0.01	-	-	0.05
**7**	*	**2-butanone, 4-phenyl-**	**148**	**C_10_H_12_O**	**0.04**	**0.16**	**0.17**	**0.29**	**0.08**	**0.12**
8	*	β-methylbenzenepropanal	148	C_10_H_12_O	-	-	-	1.07	0.21	0.31
9	○	nonanoic acid	158	C_9_H_18_O_2_	-	0.04	-	-	-	-
10	△	α-santalene	204	C_15_H_24_	0.02	-	-	-	-	-
11	△	1,4,7,-cycloundecatriene, 1,5,9,9-tetramethyl-, *Z*,*Z*,*Z*-	204	C_15_H_24_	-	-	0.04	-	-	-
12	△	humulene	204	C_15_H_24_	0.26	1.87	1.05	-	0.09	-
13	△	humulene epoxide II	220	C_15_H_24_O	0.03	-	0.06	0.09	-	-
14	△	(4*S*,4a*R*,6*R*)-4,4a-dimethyl-6-(prop-1-en-2-yl)-1,2,3,4,4a,5,6,7-octahydronaphthalene	204	C_15_H_24_	0.05	0.12	0.07	0.21	-	0.03
15	*	1,1,4,5,6-pentamethyl-2,3-dihydro-1*H*-indene	188	C_14_H_20_	0.01	0.04	0.02	-	-	-
16	△	α-curcumene	202	C_15_H_22_	0.09	0.07	-	-	-	-
17	△	β-selinene	202	C_15_H_22_	-	-	0.04	-	-	-
18	△	δ-guaiene	204	C_15_H_24_	0.02	0.05	0.05	-	0.22	-
19	△	α-bisabolene	204	C_15_H_24_	-	-	-	0.33	-	0.2
20	△	kessane	222	C_15_H_26_O	0.02	-	0.04	-	0.02	0.23
21	△	elemol	222	C_15_H_26_O	-	0.1	0.05	-	0.02	-
22	△	β-elemene	204	C_15_H_24_	-	0.1	0.09	0.1	0.04	-
23	△	furan, 3-(4,8-dimethyl-3,7-nonadienyl)-	218	C_15_H_22_O	0.17	-	-	-	-	-
**24**	**△**	**santalol, cis,*α*-**	**220**	**C_15_H_24_O**	**0.9**	**0.54**	**0.24**	**0.25**	**0.54**	**0.23**
25	△	epi-*γ*-eudesmol	222	C_15_H_26_O	0.17	-	0.18	-	-	-
**26**	**△**	**2-naphthalenemethanol, 2,3,4,4a,5,6,7,8-octahydro-a,a,4a,8-tetramethyl-, (2*R*,4a*S*,8*R*)-**	**222**	**C_15_H_26_O**	**0.11**	**0.65**	**0.25**	**0.23**	**0.06**	**0.15**
27	△	(-)-aristolene	204	C_15_H_24_	0.42	1.04	0.48	0.49	-	0.17
28	△	kusenol	222	C_15_H_26_O	-	0.13	-	-	0.49	-
**29**	**△**	**agarospirol**	**222**	**C_15_H_26_O**	**0.47**	**1.42**	**0.81**	**0.81**	**0.13**	**0.27**
30	△	guaiol	222	C_15_H_26_O	1.32	-	-	-	0.16	-
31	△	2-(4a,8-dimethyl-2,3,4,5,6,8a-hexahydro-1*H*-naphthalen-2-yl)propan-2-ol	222	C_15_H_26_O	-	3.89	2.61	3.04	-	1.3
32	△	trans-guai-11-en-10-ol	222	C_15_H_26_O	-	0.11	-	-	0.2	-
33	△	neointermedeol	222	C_15_H_26_O	0.27	0.88	-	-	0.2	-
34	△	pogostole	222	C_15_H_26_O	-	-	0.59	-	-	-
35	△	eudesma-3,11(13)-dien-12-al	218	C_15_H_22_O	0.17	0.88	0.36	-	-	-
36	△	*α*-bisabolol	222	C_15_H_26_O	0.88	-	-	-	-	-
37	△	*β*-santalol	220	C_15_H_24_O	0.49	-	-	-	-	-
38	△	farnesol	222	C_15_H_26_O	0.82	-	-	-	-	-
39	*	(E)-2-methyl-6-(p-tolyl)hept-2-en-1-yl acetate	260	C_17_H_24_O_2_	1.73	-	-	0.34	-	-
40	△	eremophilone	218	C_15_H_22_O	-	0.12	-	0.87	0.09	0.28
41	○	(E)-5-((1*S*,5*R*,8a*R*)-5-formyl-5,8a-dimethyl-2-methylenedecahydronaphthalen-1-yl)-3-methylpent-2-en-1-yl acetate	346	C_22_H_34_O_3_	-	0.78	0.5	-	0.27	-
42	○	10,12-pentacosadiynoic acid	374	C_25_H_42_O_2_	-	-	-	-	0.04	-
43	△	2-hepten-1-ol, 2-methyl-6-(4-methyl-1,4-cyclohexadien-1-yl)-, (2*Z*,6*R*)-	220	C_15_H_24_O	0.05	-	-	-	-	-
44	△	lanceol, cis	220	C_15_H_24_O	0.23	-	-	-	-	-
45	△	α-costol	220	C_15_H_24_O	-	0.28	-	0.33	0.18	-
46	△	2-naphthalenemethanol, decahydro-8-hydroxy-, alpha., alpha.,4a,8-tetramethyl	240	C_15_H_28_O_2_	0.26	2.87	1.31	-	-	-
**47**	**△**	**2(3*H*)-naphthalenone, 4,4a,5,6,7,8-hexahydro-4a,5-dimethyl-3-(1-methylethylidene)-, (4ar-cis)-**	**218**	**C_15_H_22_O**	**0.34**	**1.8**	**0.56**	**0.88**	**0.26**	**0.51**
48	△	1*H*-cyclopropa[a]naphthalene, decahydro-1,1,3a-trimethyl-7-methylene-, [1a*S*-(1a.alpha.,3a.alpha.,7a.beta.,7b.alpha.)]-	204	C_15_H_24_	-	-	-	-	0.01	-
49	*	methyl isocostate	248	C_16_H_24_O_2_	0.12	-	-	-	-	-
50	△	proximadio	240	C_15_H_28_O_2_	-	-	0.37	0.28	0.06	0.14
51	△	7-hydroxy-9-(hydroxymethyl)-1,4,9-trimethyl-2,4,5,7,8,8a-hexahydro-4,7-methanoazulen-6(1*H*)-one	250	C_15_H_28_O3	-	-	-	0.69	-	-
52	○	[7-(2-hydroxypropan-2-yl)-4a-methyl-1-methylidene-2,3,4,5,6,7,8,8a-octahydronaphthalen-2-yl] acetate	280	C_17_H_28_O_3_	0.99	-	-	1.35	0.16	0.56
**53**	**△**	**arctiol**	**238**	**C_15_H_26_O_2_**	**0.25**	**1.51**	**0.74**	**0.66**	**0.15**	**0.27**
54	△	azuleno[6,5-b]furan-2(3*H*)-one, (3*R*,3a*R*,4a*S*,5*R*,9a*S*)-3,5,8-trimethyl-3a,4,4a,5,6,7,9,9a-octahydroazuleno[6,5-b]furan-2(3*H*)-one	234	C_15_H_22_O_2_	0.17	1.21	1.17	0.45	-	0.23
55	△	caryophyllene oxide	220	C_15_H_24_O	0.84	-	-	-	-	-
56	△	bicyclo[2.2.1]heptan-2-ol, 7-[(3*E*)-5-hydroxy-4-methyl-3-pentenyl]-1,7-dimethyl-, (1*S*,2*R*,4*S*,7*R*)-	238	C_15_H_26_O_2_	0.4	-	-	-	-	-
57	*	1,5-diphenyl-3-pentanone	238	C_17_H_18_O	0.26	0.58	0.58	-	-	-
58	○	1-phenanthrenecarboxaldehyde, 7-ethenyl-1,2,3,4,4a,4b,5,6,7,8,10,10a-dodecahydro-1,4a,7-trimethyl-, (1*R*,4a*R*,4b*S*,7*S*,10a*R*)-	286	C_20_H_30_O	0.44	-	0.4	-	-	-
59	*	dibenzo[a,h]cyclotetradecene, 2,3,11,12-tetraethenyl-1,2,3,4,5,6,7,8,9,10,11,12,13,14,15,16,17,18-octadecahydro-, (2R*,3S*,4*Z*,9*Z*,11R*,12S*)-	404	C_30_H_44_	0.31	-	-	-	-	-
60	△	viridiflorol	222	C_15_H_26_O	0.32	0.37	0.11	-	-	-
61	△	carisson	236	C_15_H_24_O_2_	0.16	-	-	-	-	-
62	○	9-octadecenoic acid	282	C_18_H_34_O_2_	-	-	0.26	-	-	-
**63**	*	**1-penten-3-one, 1,5-diphenyl-**	**236**	**C_17_H_16_O**	**0.12**	**0.46**	**0.44**	**0.21**	**0.09**	**0.75**
64	*	3-phenylbutylaldehyde	148	C_10_H_12_O	0.23	0.86	1.05	1.05	-	0.2
65	*	3-hydroxy-1,5-diphenyl-1-pentanone	254	C_17_H_18_O_2_	-	0.08	0.12	0.21	-	-
**66**	**☆**	**2-phenethyl-4*H*-chromen-4-one**	**250**	**C_17_H_14_O_2_**	**20.24**	**12.69**	**21.26**	**24.62**	**41.42**	**38.81**
**67**	**☆**	**6-methoxy-2-phenethyl-4*H*-chromen-4-one**	**280**	**C_18_H_16_O_3_**	**3.92**	**4.51**	**7.29**	**1.59**	**0.07**	**10.91**
**68**	**☆**	**2-(4-methoxyphenethyl)-4*H*-chromen-4-one**	**280**	**C_18_H_16_O_3_**	**23.53**	**1.03**	**8.24**	**33.95**	**35.26**	**1.58**
**69**	**※**	**squalene**	**410**	**C_30_H_50_**	**0.1**	**0.15**	**0.24**	**0.39**	**0.15**	**0.13**
70	☆	6-methoxy-2-(4-methoxyphenethyl)-4*H*-chromen-4-one	310	C_19_H_18_O_4_	0.27	0.1	0.31	-	-	-
71	☆	6,7-dimethoxy-2-phenethyl-4*H*-chromen-4-one	310	C_19_H_18_O_4_	7.82	1.01	3.74	4.87	-	0.75
72	☆	6,7-dimethoxy-2-(4-methoxyphenethyl)-4*H*-chromen-4-one	340	C_20_H_20_O_5_	0.93	-	0.35	0.54	-	-
73		stigmasterol	412	C_29_H_48_O	0.29	0.12	0.39	0.83		0.06

Sesquiterpenes △; aromatic compounds *; aliphatic compounds ○; 2-(2-phenylethyl)chromones ☆; triterpenes ※; common components extracted by the SFE-CO_2_ method (**Black bold font**). The value of each component in C1 to C6 was the average of three samples.

**Table 2:** Seven distinguished violate markers of agarwood oils between the native *A. sinensis* and ‘Qi-Nan’ subspecies.

Sort	Compounds Markers	VIP Value
1	2-phenethyl-4*H*-chromen-4-one	5.32
2	2-(4a,8-dimethyl-2,3,4,5,6,8a-hexahydro-1*H*-naphthalen-2-yl)propan-2-ol	1.84
3	6,7-dimethoxy-2-phenethyl-4*H*-chromen-4-one	1.76
4	3-phenylbutylaldehyde	1.13
5	2-naphthalenemethanol, decahydro-8-hydroxy-, alpha, alpha, 4a,8-tetramethyl	1.11
6	agarospirol	1.02
7	3a,4,4a,5,6,7,9,9a-octahydro-3,5,8-trimethyl-, (3*R*,3a*R*,4a*S*,5*R*,9a*S*)-	1.01

**Table 3:** Source information on the 6 planting area samples.

No.	Sources	Species
C1	Nantou, Taiwan (TN)	*A. sinensis*
C2	Maoming, Guangdong (GM)	*A. sinensis*
C3	Chuhai, Guangdong (GC)	*A. sinensis*
C4	Nanning, Guangxi (XN)	*A. sinensis*
C5	Huizhou, Guangdong (GH)	*A. sinensis* ‘Qi-Nan’
C6	Huidong, Guangdong (GD)	*A. sinensis* ‘Qi-Nan’

## Data Availability

All data generated or analyzed during this study are included in this published article.

## Abbreviations

Supercritical CO_2_ extraction, SFE-CO_2_; agarwood oils, AOs; gas chromatography–mass spectrometry, GC–MS; principal components analysis, PCA; partial least squares discriminant analysis, PLS-DA; orthogonal partial least squares discriminant analysis, OPLS-DA.
